# Progressed Multivessel Spontaneous Coronary Artery Dissection That Naturally Healed in a Male Patient with Non-ST Segment Elevation Myocardial Infarction

**DOI:** 10.1155/2016/4109496

**Published:** 2016-05-30

**Authors:** Tatsuo Haraki, Ryota Uemura, Shin-ichiro Masuda, Takeshi Lee

**Affiliations:** Department of Cardiology, Saitama Eastern Cardiovascular Hospital, 3187-1 Osawa, Koshigaya, Saitama 343-0025, Japan

## Abstract

Spontaneous coronary artery dissection (SCAD) is a rare condition that may have a serious outcome because of acute coronary syndrome. The condition especially affects young women. We evaluated a middle-aged male patient with a non-ST segment elevation myocardial infarction caused by multivessel SCAD. The SCAD had occurred in the distal right coronary artery (RCA), the mid left anterior descending artery (LAD), and the distal LAD at the same time. His culprit lesion was in the distal RCA, but the SCAD had progressed more proximally within the RCA 12 days later with no clinical symptoms. We treated the mid LAD with implantation of a drug-eluting stent on admission and the SCAD had not progressed 12 days later. Moreover, the SCAD in the distal RCA and distal LAD healed spontaneously 12 days later. He had no recurrent attack, and all SCAD lesions of the RCA and LAD had completely healed 6 months later. Given that SCAD appears in various forms over the clinical course, a strategy of intervention needs careful consideration.

## 1. Introduction

Spontaneous coronary artery dissection (SCAD) is a rare condition that predominantly affects young healthy women. It can sometimes cause serious outcomes because of sudden death and acute coronary syndrome (ACS). The clinical features that give rise to suspicion of SCAD are (a) myocardial infarction (MI) in young women, especially those aged ≤50 years, (b) the absence of traditional cardiovascular risk factors, (c) little or no evidence of typical atherosclerotic lesions in the coronary arteries, (d) a peripartum condition, (e) a history of fibromuscular dysplasia (FMD), and (f) either emotional or physical (intensive exercise) precipitating stress events [1–3]. It has been reported that the frequency of ACS caused by multivessel SCAD was relatively high (observed in approximately 20% of cases), but the effects of interventional in-hospital treatment and long-term outcomes remain controversial [4–6]. We report on a case of a middle-aged male patient with an inferior non-ST segment elevation myocardial infarction (NSTEMI) caused by multivessel SCAD. The SCAD in the distal right coronary artery (RCA) progressed more proximally but finally healed naturally with conservative treatment.

## 2. Case Presentation

A 49-year-old male had felt severe chest pain accompanied by a cold sweat during his evening work of garbage collection. The symptoms continued until the next morning when he consulted our hospital as a walk-in patient. He had no coronary risk factors but he had been taking a proton-pump inhibitor for regurgitant esophagitis for a few years. An electrocardiogram demonstrated abnormal Q waves in leads II, III, and aVF without an ST-T change. A chest X-ray showed no lung congestion. His CK (879 U/L), CK-MB (87 U/L), and troponin T (1.8 ng/mL) levels were all elevated. The echocardiography demonstrated hypokinesis in the inferoseptal wall of the left ventricle. Acute inferior NSTEMI was the suggested diagnosis and we started a loading dual antiplatelet therapy (DAPT) of 200 mg of aspirin and 20 mg of prasugrel, 96 mg/d of nicorandil, and 10000 units/d of heparin under a continuous intravenous injection. We also performed an emergent coronary angiogram (CAG).

**Figure 1:**
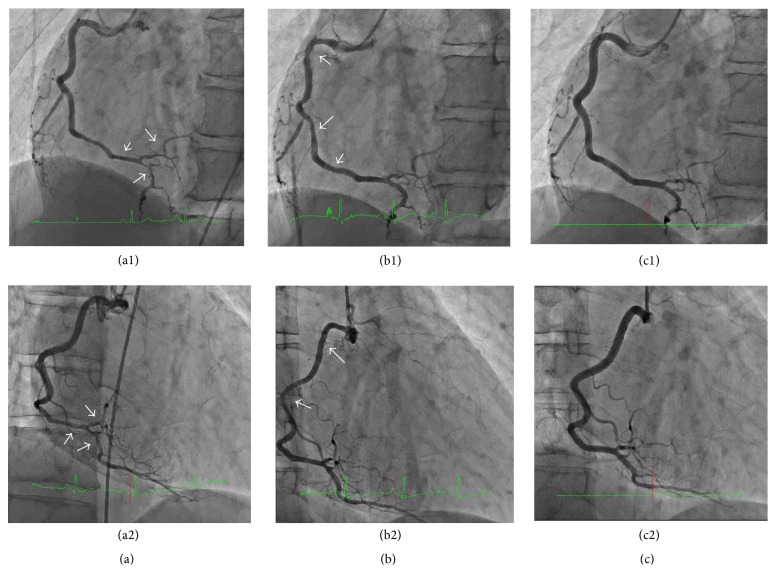
Time course of a coronary angiogram of the right coronary artery (RCA). (a) MI onset; (b) 12 days after the onset of the MI; (c) 6 months after the onset of the MI. 1: LAO view; 2: RAO view.

**Figure 2:**
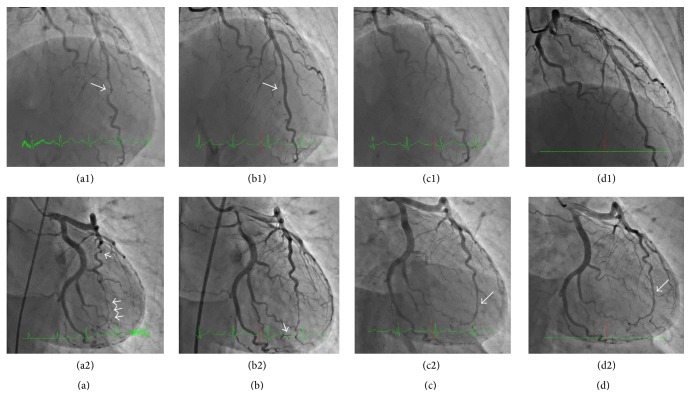
Time course of a coronary angiogram of the left coronary artery (LCA). (a) MI onset; (b) after implantation of a zotarolimus-eluting stent in the mid LAD; (c) 12 days after the onset of the MI; (d) 6 months after the onset of the MI. 1: RAO cranial view; 2: straight caudal view.

**Figure 3:**
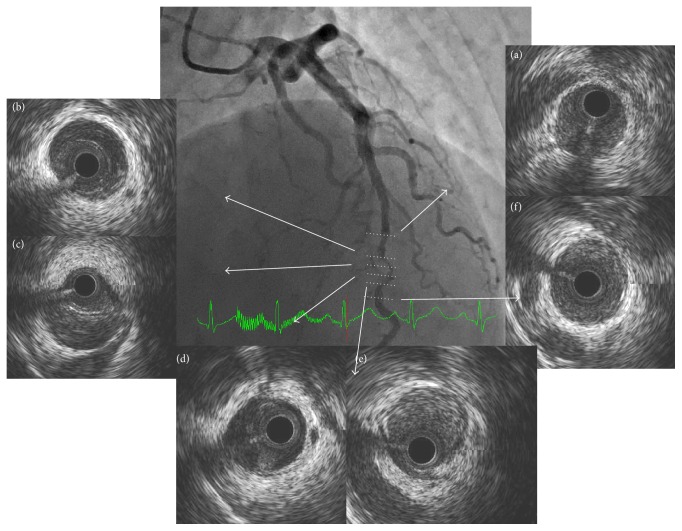
IVUS findings in the mid LAD on admission. Double lumen and an intramural hematoma were found (b–e). CAG: straight cranial view.

The CAG showed that the RCA had diffuse stenosis in the distal RCA which was not dilated by an isosorbide dinitrate (ISDN) intracoronary injection (Figures 1(a1) and 1(a2)). The left circumflex artery (LCX) was normal, but the left anterior descending artery (LAD) was tortuous and mid LAD was focally near occlusion, identified by contrast staining (Figure 2(a1)). The more distal LAD showed diffuse severe stenosis which again did not dilate following an ISDN injection (Figure 2(a2)). We performed an intracoronary ultrasound (IVUS) near the occluded lesion in the mid LAD because his symptoms were not relieved. Double lumen and an intramural hematoma were found (Figures 3(b)–3(e)), but there were no atherosclerotic plaques. These findings were compatible with SCAD. His symptom worsened after the IVUS so we performed a percutaneous coronary intervention (PCI) in the mid LAD. The dissection spread after a conventional balloon angioplasty, so we implanted a 2.5 × 26 mm zotarolimus-eluting stent (Medtronic, Inc., Santa Rosa, CA, USA) in the mid LAD. The mid LAD was well dilated and received good blood flow after stent implantation (Figure 2(b1)), but the more distal LAD was still severely narrowed with contrast staining (Figure 2(b2)). Cardiac rehabilitation was performed after the PCI and he had no recurrent chest pain or ischemia.

**Figure 4:**
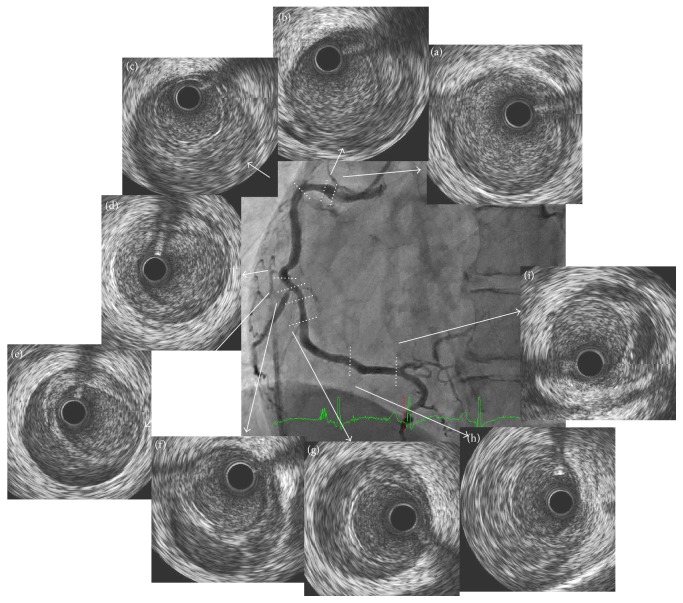
IVUS findings in the RCA 12 days after the onset of the MI. An intramural hematoma from the proximal to the distal RCA and double lumen were observed in spots (b–i). CAG: LAO view.

**Figure 5:**
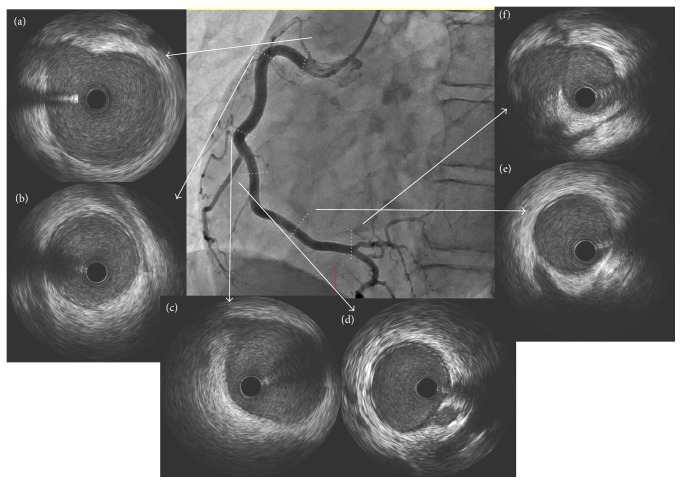
IVUS findings in the RCA 6 months after the onset of the MI. Spontaneous healing of the SCAD was observed in the whole RCA. CAG: LAO view.

We performed a follow-up CAG 12 days after the MI onset. The stenting site in the mid LAD was patent, and the distal LAD was dilated compared with that on admission (Figure 2(c2)). The distal RCA was also dilated compared with that on admission, but the area proximal to the mid RCA showed progression of luminal narrowing (Figures 1(b1) and 1(b2)). The IVUS confirmed an intramural hematoma proximal to the distal RCA and double lumen was observed in spots (Figures 4(b)–4(i)). The SCAD had tended to spread to a more proximal lesion of the RCA 12 days after the onset of the MI. In contrast, the dissection had not spread to the proximal lesion in the stent implanted LAD, and the distal LAD lesion was healing. Although we had selected a conservative therapy for the RCA, he had no recurrence over the next 6 months. Spontaneous healing of the SCAD was observed in both the whole RCA (Figures 1(c1) and 1(c2)) and the LAD (Figures 2(d1) and 2(d2)), as confirmed by a CAG and by an IVUS (Figure 5) 6 months after the onset of the MI.

## 3. Discussion

We evaluated a case of a middle-aged male patient with NSTEMI caused by multivessel SCAD. The SCAD was observed in the distal RCA and in the mid and distal LAD at the same time on admission. The SCAD of the distal RCA had spread more proximally 12 days later, but it finally healed naturally with conservative treatment. Our patient had an absence of traditional cardiovascular risk factors, but he had participated in intensive exercise (garbage collection) at the onset of the SCAD. A renal angiogram showed no findings of FMD.

SCAD is caused by an intraluminal hemorrhage or intimal tear, but an angiographic diagnosis is difficult because diffuse stenosis is common. An angiographic SCAD diagnosis is categorized as Type 1 (evident of arterial wall stain) in 29.1%; Type 2 (diffuse stenosis of varying severity) in 67.0%; and Type 3 (mimic atherosclerosis) in 3.9% [7]. Therefore, IVUS and optical coherence tomography (OCT) are established and valuable tools for the diagnosis of SCAD [8].

Although his culprit lesion was considered to be in the distal RCA, we treated the mid LAD with implantation of a drug-eluting stent (DES) at the onset of MI. At that time, we performed IVUS at near-occluded lesion in the mid LAD because his symptoms were not relieved. IVUS findings could show that SCAD had occurred in this patient. In contrast, we did not perform an evaluation by IVUS in the RCA, because his distal RCA with diffuse, severe narrowing seemed to be unsuitable for IVUS study. Interestingly, his RCA showed mimic atherosclerotic changes from the proximal to mid RCA 12 days later. We speculated that the SCAD had naturally spread more proximally in the RCA. In contrast, the SCAD lesion in the mid LAD, which we treated with stent implantation, had not spread more proximally in the LAD. A progressive SCAD was reported in 10% of SCAD patients and occurred generally within seven days [9]. In contrast, spontaneous healing was found in most patients following conservative treatment confirmed by a CAG >26 days later [7].

PCI for SCAD was associated with high rates of technical failure, even in those who presented with a preserved vessel flow, while a strategy of conservative management with prolonged observation was associated with good early and long-term outcomes [9]. One reason for the high technical failure rates of PCI may be because of less frequent usage (18%) of imaging modality compared with an IVUS or OCT [9]. Imaging modality could distinguish the guidewire position from the true to false lumen as well as the distribution of the SCAD. We achieved full coverage in the mid LAD of SCAD lesion with stent implantation by IVUS guidance. Technical success was achieved by full coverage of the SCAD lesion with stent implantation [10], or by cutting balloon angioplasty which could reduce an intramural hematoma with OCT guidance [11], and by spot stenting which could cover the entry point of intimal tear with OCT guidance [12]. These procedures using imaging modality may prevent further progression of SCAD and lead to higher success rate of PCI.

We selected zotarolimus-eluting stent (ZES) implantation in the mid LAD, because the culprit of luminal size was relatively small (reference vessel diameter of 2.23 mm). Clinical trial using the ZES in the small coronary vessels demonstrated that the suppression of the neointimal hyperplasia, lower in-stent late lumen loss, and good long-term clinical outcomes were observed in the Japanese patients [13]. However, it is reported that stent malapposition because of the resorption of intramural hematoma was observed by OCT in several patients with SCAD who were treated with DES [14]. If DES is selected for the treatment of the SCAD lesions, the delayed healing in the lesion with major dissection should be considered. In addition, long-term DAPT may disturb the thrombus formation within the false lumen. Incomplete stent apposition, which was observed by CAG as a peristent contrast staining (PSS) after sirolimus-eluting stent implantation, is associated with very late stent thrombosis [15]. Therefore, repeat CAG as well as OCT and/or IVUS study should be recommended before the cessation of DAPT, if DES treatment is chosen for the SCAD lesions.

In contrast, another report indicated that a more conservative strategy was associated with better in-hospital outcomes compared with revascularization therapy [16]. However, no significant differences were observed in the long-term outcomes. This could be explained by a high recurrent SCAD rate of 10%–27% at 2–5 years' follow-up [7, 9], which included new lesions as well as initially diseased vessels [9, 17].

Thus, a more conservative therapy is commonly recommended unless there are features of ongoing/recurrent ischemia, hemodynamic and electrical instability, and left main or proximal segment LAD, LCX, and RCA dissections [1–5]. We selected stent implantation at the mid LAD and conservative treatment at the RCA of the SCAD lesion. There is a possibility that stent implantation may prevent progression of the SCAD at the acute phase. However, the SCAD progression in the RCA also finally healed with conservative treatment, which indicated that it may be preferable to select conservative treatment when the patient is clinically stable. Moreover, it is important to avoid the origin and trigger after the onset of SCAD.

## 4. Conclusion

We evaluated a male patient with NSTEMI caused by multivessel SCAD. The SCAD of the distal RCA progressed to a proximal lesion 12 days later. A stent implanted in the mid LAD did not progress to a proximal lesion 12 days later, and the whole RCA and LAD were finally healed 6 months after the onset of the MI. Progressive SCAD was observed 12 days after the onset of the MI, but spontaneous healing was observed in both lesions with conservative and stent implantation treatment 6 months later. Considering that SCAD may appear in variable forms over the clinical course, a careful consideration of whether to use conservative or interventional therapy treatment options is required. The use of imaging modality, such as IVUS or OCT, should be considered when PCI is chosen at the time of acute phase as well as follow-up period.
